# A new approach to mixing techniques for enhanced performance in automated sample preparation

**DOI:** 10.1155/S1463924695000253

**Published:** 1995

**Authors:** François Qian, Eric Vérette, Atika El-Sayed

**Affiliations:** Gilson Medical Electronics (France) S.A. 72 rue Gambetta B.P. 45 Villiers-le-Bel 95400 France

## Abstract

In the automation of sample dilution or derivatization, the performance of the mixing technique employed when adding solvents or reagents to samples is critical. This paper presents a newly developed mixing method, based on conventional aspiration and dispensing of liquid techniques, but which considerably improves the precision of mixing. The paper discusses the results of a comparison of the technique with other methods and describes the application of the technique to several different types of sample solutions, including a highly concentrated glucose solution. The mixing technique was performed on a Gilson XL Sampling Injector, with a 1/25 dilution of a paraben solution in 2 ml vials to give relative standard deviations of 0.2 to 0.3% (N =10).


Journal of Automatic Chemistry, Vol. 17, No. 5 (September–October 1995), pp. 169–171

### A new approach to mixing techniques for enhanced performance in automated sample preparation

**François Qian, Eric Vérette and Atika El-Sayed**
*Gilson Medical Electronics (France) S.A., 72 rue Gambetta, B.P. 45, 95400 Villiers-le-Bel, France*

*In the automation of sample dilution or derivatization, the performance of the mixing technique employed when adding solvents or reagents to samples is critical. This paper presents a newly developed mixing method, based on conventional aspiration and dispensing of liquid techniques, but which considerably improves the precision of mixing. The paper discusses the results of a comparison of the technique with other methods and describes the application of the technique to several different types of sample solutions, including a highly concentrated glucose solution. The mixing technique was performed on a Gilson XL Sampling Injector, with a 1/25 dilution of a paraben solution in 2 ml vials to give relative standard deviations of 0·2 to 0·3% (N = 10).*

### Introduction

To meet the demands on analytical laboratories for higher throughputs with a greater reproducibility, the automation of liquid sample preparation procedures is increasingly becoming a necessity. However, whether performing dilution or reagent addition, such as in derivatization, the weak link in many automated sample preparation protocols is the mixing step. The precision of mixing contributes directly to the total performance of an analytical method [1, 2].

The different mixing techniques currently available for use with autosamplers or sample processors all have limitations. The techniques of stirring or vortexing are difficult to automate without risk of carry-over; mixing by air is unsuitable for air-sensitive samples, volatile samples or samples, such as protein solutions like sera, with high foaming properties; and conventional mixing by aspirating and dispensing of liquids is not always sufficient, particularly for viscous liquids or high dilution ratios.

This paper presents a new and highly efficient mixing method—'stepping mixing'. The method permits dilution, mixing and injection of samples with excellent relative standard deviations (RSDs), and is suitable for a wide range of dilution ratios and viscous samples.

### Method

The new mixing method was developed on a Gilson Series XL Sampling Injector, and applied to the dilution of methyl- and propyl-paraben solutions in 2 ml flat-bottom, capped glass vials.

### *Principle*

The stepping mixing method is based on the conventional technique of aspirating and dispensing of liquids, but with two significant developments:

(1) The total volume to be mixed ($Vol_{total}$) is aspirated in one step, but is then dispensed in several steps (for example $Vol_1$ and $Vol_2$) with different dispensing flow rates.
(2) The flow rates of the aspirating and the different dispensing steps are optimized according to the characteristics of the solution to be mixed.

### *Experimental conditions*

(1) HPLC Instrumentation from Gilson: 307 Pump (5 SC pump head), 118 UV/Visible Detector, with 715 System Controller Version 1.21 and 506C Interface.

(2) Sample: 1 mg/ml each of methyl- and propyl-paraben (Sigma) in 100% methanol (Carlo Erba).

(3) Dilution ratio: 1/25 (32 µl sample solution diluted with 768 µl diluent).

(4) Diluent: same as mobile phase.

(5) Mobile phase: acetonitrile–water (55:45) from J. T. Baker, flow rate at 1 ml/min.

(6) UV detection: at 265 nm (0·8 AUFS).

(7) Column: Spherisorb ODS 2, 5 µm (150 × 4·6 mm) from Shandon.

(8) Injection volume: 20 µl with total loop filling (loop filling coefficient of 5).

(9) Injection sequence: 5 injections per vial. RSDs were calculated from a series of 10 injections.

### Results

### *Performance of mixing and injection*

The chromatographic results for sample solutions following dilution using stepping mixing, demonstrate the efficiency of mixing within a vial and in different vials (see table 1). The RSD values represent the total imprecision for both the analysis and sample preparation. A second run of five series of 10 injections performed on another day gave comparable results, with an RSD of 0·3%.

### *Linearity of dilution*

The performance of stepping mixing was evaluated for sample solutions prepared with different dilution ratios (see table 2). The calibration graph for methyl-paraben over the range of dilution ratios studied is shown in figure 1.

0142-0453/95 $10.00 © 1995 Taylor & Francis Ltd.

*Table 1. Injection series for samples diluted 25-fold using stepping mixing method.*

| Sample name | Loop No. | Mean area | SD | RSD | No. of injections |
|---|---|---|---|---|---|
| Methyl-paraben | 1–10 | 87 299 | 238 | 0·3% | 10 |
| | 11–20 | 87 609 | 104 | 0·1% | 10 |
| | 21–30 | 87 446 | 117 | 0·1% | 10 |
| | 31–40 | 87 322 | 249 | 0·3% | 10 |
| | 41–50 | 87 445 | 266 | 0·3% | 10 |
| | 1–50 | 87 424 | 227 | 0·3% | 50 (*10 vials*) |
| Propyl-paraben | 1–10 | 76 118 | 223 | 0·3% | 10 |
| | 11–20 | 76 375 | 91 | 0·1% | 10 |
| | 21–30 | 76 256 | 112 | 0·1% | 10 |
| | 31–40 | 76 143 | 236 | 0·3% | 10 |
| | 41–50 | 76 220 | 214 | 0·3% | 10 |
| | 1–50 | 76 222 | 200 | 0·3% | 50 (*10 vials*) |

*Table 2. Injection series for samples prepared at various dilution ratios using stepping mixing method.*

| Sample name | Dilution ratio | RSD | No. of injections |
|---|---|---|---|
| Methyl-paraben | 1/10 | 0·3% | 10 |
| | 1/25 | 0·2% | 10 |
| | 1/50 | 0·2% | 10 |
| | 1/100 | 0·2% | 10 |
| | 1/250 | 0·3% | 10 |
| Propyl-paraben | 1/10 | 0·2% | 10 |
| | 1/25 | 0·2% | 10 |
| | 1/50 | 0·2% | 10 |
| | 1/100 | 0·2% | 10 |
| | 1/250 | 0·5% | 10 |

*Table 3. Comparison of different mixing techniques.*

| Mixing method | RSD | Remarks |
|---|---|---|
| Stepping | 0·2–0·3% | Precise<br>Rapid<br>Simple |
| Air | 0·4–0·6% | Simple<br>Risk of oxidation<br>Risk of foaming |
| Ultrasonic | 0·4–1·0% | Risk of over-heating of sample from ultrasonic bath<br>Noise pollution<br>More than 3 min mixing required for RSD below 1·0%<br>Additional accessories required |
| Mechanical shaking | 0·7–1·0% | More than 10 min mixing required for RSD below 1·0%<br>Additional accessories required |

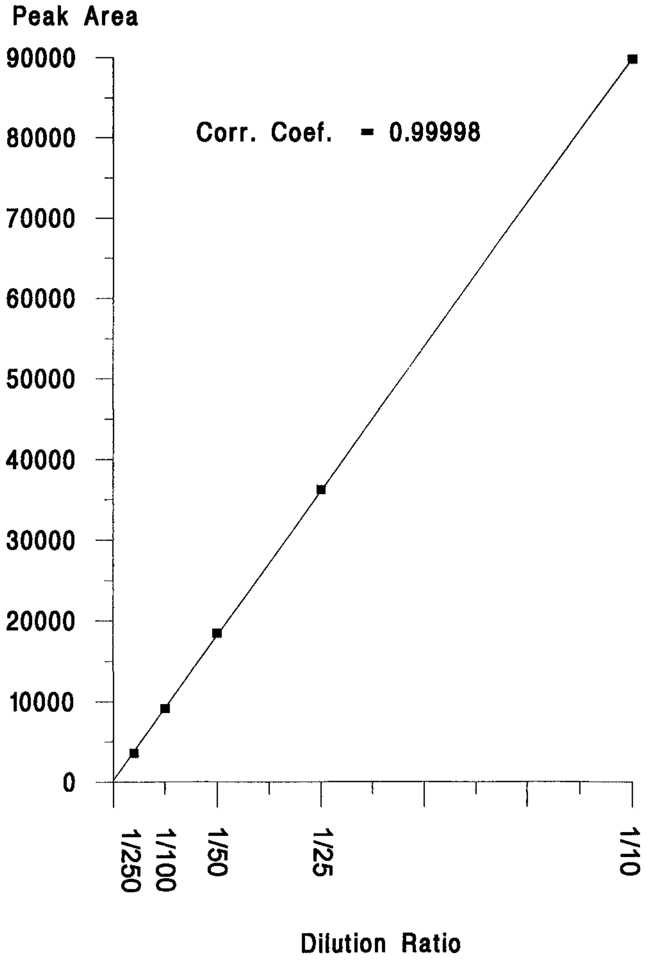

*Figure 1. Linearity curve for methyl-paraben sample solutions prepared at various dilution ratios using stepping mixing method.*

*Table 4. Results for three consecutive injection series for β-D-(+)-glucose solution (200 g/l), diluted 10-fold with water using stepping mixing method.*

| Sample name | Dilution ratio | RSD | No. of injections |
|---|---|---|---|
| β-D(+)-glucose | 1/10 | 0·7% | 10 |
| | 1/10 | 0·6% | 10 |
| | 1/10 | 0·6% | 10 |

*Comparison with other mixing techniques*

The stepping mixing method was compared with other mixing techniques, also performed on paraben samples using a 233 XL Sampling Injector, with a 1/25 dilution ratio and identical chromatographic conditions (see table 3).

*Mixing of concentrated glucose solution*

Stepping mixing provided precise mixing in the dilution of a highly concentrated glucose solution (see table 4).

### Conclusion

Stepping mixing is based on the technique of aspirating and dispensing of liquids. By dispensing the mixing volume in several steps at different dispensing flow rates, a more efficient and precise mixing effect is achieved.

With use of the stepping mixing method, homogenous paraben solutions were prepared with a 25-fold dilution and injected onto an HPLC system to give RSDs of 0·3% ($N = 10$). The calibration graph was rectilinear over a dilution range of 1/10 to 1/250. The method was applied in the dilution of several different types of sample solutions, including a highly concentrated glucose solution.

Comparison with other autosampler mixing techniques shows that the stepping mixing method offers fast, high precision mixing, which is easy to implement and does not require additional accessories.

### References

1. Lindon, M. J., *Journal of Chromatography*, **624** (1992), 3–9.
2. Nau, V., White, L. and Wu, R. *International Laboratory*, **8** (1992), 2–4.

